# Isolated Crohn's Disease of the Appendix Presenting as Acute Appendicitis in a 60-Year-Old South Asian Female: A Case Report, Review of Literature, and Follow-Up Recommendations

**DOI:** 10.1155/2019/5285417

**Published:** 2019-10-13

**Authors:** Pamathy Gnanaselvam, Dhanushka N. Weerakoon, W. A. M. Wijayasuriya, Vishva Samidi Mohottala, B. M. E. S. Sinhakumara, Umesh Jayarajah, Anura S. K. Banagala

**Affiliations:** Department of Surgery, National Hospital of Sri Lanka, Colombo, Sri Lanka

## Abstract

The isolated appendiceal Crohn's disease without preceding bowel symptoms is a rare phenomenon, especially in older patients. In this case report, we present a 60-year-old female with isolated appendiceal Crohn's disease presenting with acute appendicitis. She presented with classical features of appendicitis with elevated inflammatory markers. She underwent an appendectomy which showed an excessively swollen, oedematous, and reddish appendix with swelling extending to the base of the caecum. Histological evaluation was suggestive of Crohn's disease, and subsequent colonoscopy was unremarkable. Following appendectomy, she was asymptomatic without any recurrence of disease. The atypical morphological appearance of the appendix should raise suspicion of Crohn's disease. This case highlights the importance of histopathological analysis of the specimen, especially in abnormal clinical findings. The prognosis of such patients seems to be good, and additional treatment is rarely needed.

## 1. Introduction

Crohn's disease is a complex chronic inflammatory bowel disorder characterized by a transmural inflammatory reaction and noncaseating granulomas [1]. It may involve all parts of the gastrointestinal tract from the mouth to the anus, commonly occurring in the ileum and colon [2, 3]. Appendiceal Crohn's disease is infrequent but has been well documented in the literature with an incidence of 0.2-0.55%; however, it occurs more frequently in young individuals [3]. The isolated involvement of the appendix is uncommon, without any preceding bowel symptoms, especially in older patients [4]. In this case report, we present a 60-year-old female with isolated appendiceal Crohn's disease presenting as acute appendicitis.

## 2. Case Presentation

**Figure 1:**
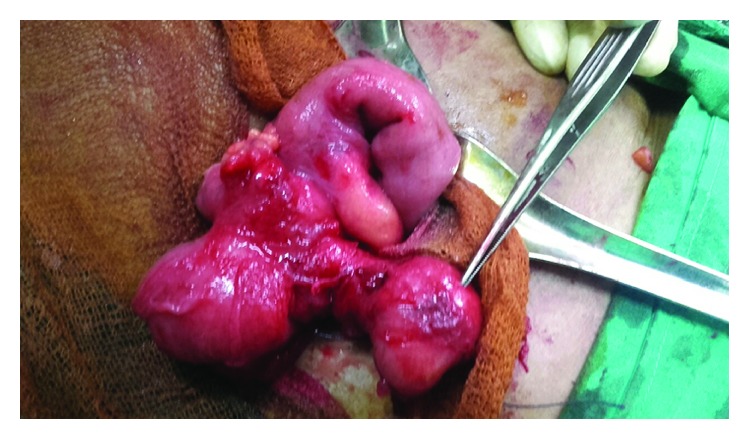
An excessively swollen, oedematous, and reddish appendix with swelling extending to the base of the caecum. The swelling was more prominent at the tip of the appendix (shown by the instrument) giving a globular shape.

**Figure 2:**
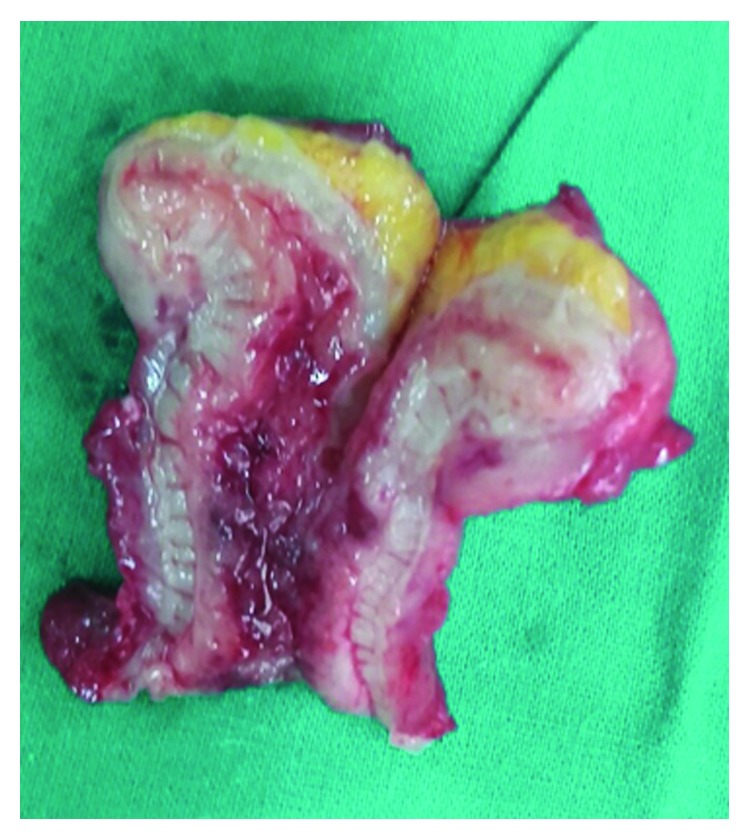
Cut section of the appendix showing an oedematous wall with significant mural thickening.

A 60-year-old female patient who was previously healthy presented with a history of right iliac fossa pain for 3 days duration. It was a continuous pain, cramping in nature, with mild fever and anorexia. There were no other associated urinary or gastrointestinal symptoms. There was no history suggestive of inflammatory bowel disease, and no systemic manifestations of Crohn's disease, such as skin manifestations, arthralgia, or uveitis, were noted. Family history was unremarkable. The patient was acutely ill, and abdominal examination revealed a right iliac fossa tenderness with no rebound tenderness or guarding. There was no palpable mass. Biochemical evaluation showed increased white blood cells—20.1 × 10^9^/litre with neutrophil predominance (91%). C-reactive protein was elevated—120 mg/litre. Due to the worsening of symptoms, a clinical diagnosis of acute appendicitis was made and open appendectomy was performed through a Lanz incision. An excessively swollen, oedematous, and reddish appendix was noted with swelling extending to the base of the caecum. The swelling was more prominent at the tip of the appendix giving a globular shape (Figures 1 and 2). The rest of the caecum and terminal ileum appeared normal. The histology showed a transmural acute inflammatory infiltrate consisting of neutrophils, lymphocytes, and plasma cells with eosinophils. Fissures lined by vascular granulation tissue extending through the muscle wall into the subcutaneous fat were seen. Multiple noncaseating, nonconfluent granulomas composed of epitheloid histiocytes were noticed. There was no evidence of caseating type necrosis or carcinoid tumour seen. Stains for acid-fast bacilli were negative. Thus, the histological findings were consistent with Crohn's disease.

A colonoscopy was performed up to the distal ileum. The mucosa appeared normal except a small aphthous ulcer at the transverse colon. Multiple biopsies were taken from the lesion, caecum, and distal ileum, which were unremarkable.

Following surgery, there was complete relief of symptoms, and therefore, pharmacological treatment was not initiated. There were no other manifestations related to Crohn's disease. She remained symptom-free at the 6-month follow-up with no evidence of disease recurrence.

## 3. Discussion

Meyerding and Bertram reported the first isolated appendiceal Crohn's disease in 1953 [5]. Since then, many reports and reviews have been described in literature. The incidence of appendiceal Crohn's disease is variable and is generally described as 0.2-0.55% [3, 4]. Appendiceal Crohn's disease is usually seen in the young; however, the presenting age may range between 10 and 51 years [3, 4, 6]. It generally has a male preponderance [3, 4, 6]. The prevalence of inflammatory bowel disease and Crohn's disease is low in the South Asian region compared to the Western world. Furthermore, the occurrence of isolated appendiceal Crohn's disease is very rare in South Asia [7, 8]. We described a rare presentation of isolated appendiceal Crohn's disease as acute appendicitis in an older South Asian female.

Appendiceal Crohn's disease has variable clinical presentation. The commonest presentation is acute right lower quadrant pain in a young patient which is usually diagnosed and treated as acute appendicitis [3, 4]. However, around 25% of patients have other associated symptoms of Crohn's disease such as chronic abdominal pain and altered bowel habits [3]. Furthermore, the symptoms may be longstanding and recurrent compared with the usual presentation of acute suppurative appendicitis. Therefore, Crohn's disease of the appendix should be suspected in patients who present with an unusual clinical course, especially in patients who present with symptoms over a week [3]. Our patient presented following a history of right lower quadrant pain for a short duration (3 days) without any preceding symptoms. Furthermore, the first presentation of Crohn's disease in an older female is unusual. In such cases, visualisation of the colon and distal ileum or imaging is mandatory to look for colonic malignancies and tuberculosis. Our patient did not have any significant findings on endoscopy, and serial biopsies were unremarkable.

Macroscopically, appendiceal Crohn's disease presents with an enlarged, oedematous appendix with thickened appendiceal wall and periappendiceal fibrous adhesions [3]. Characteristic microscopic features include classic features of Crohn's disease such as noncaseating granulomas, transmural chronic inflammation, lymphoid aggregates, muscular hypertrophic changes, and fibrous reaction of the appendiceal wall [3]. Our patient also showed classical macroscopic and microscopic features compatible with a diagnosis of Crohn's disease.

Several case series and reports have described the diagnosis of appendiceal Crohn's disease following appendectomy for acute appendicitis (Table 1 [3, 4, 6, 9–13]). Of those, the majority were treated only with appendectomy and subsequent colonoscopy did not reveal concomitant disease in the lower gastrointestinal tract. Two studies looked for presence of TB and found to be negative [3, 10]. The routine use of pharmacological treatment has not been documented. Long-term follow-up did not reveal any recurrences in the majority.

Therefore, based on the above reports, appendectomy alone is sufficient in the majority for isolated appendiceal Crohn's disease. The prognosis of appendiceal Crohn's disease seems to be favourable with very low recurrence rate compared with Crohn's disease involving other parts of the colon [3, 6]. The postoperative colonoscopy is important in excluding concomitant ileocolonic disease. Investigations may be performed to exclude tuberculosis in highly prevalent regions. The long-term follow-up may comprise of lower gastrointestinal endoscopy for symptomatic patients, and pharmacological treatment may be avoided in isolated asymptomatic appendiceal Crohn's disease. In our patient, appendectomy was potentially curative resulting in relief of symptoms. Furthermore, there was no clinical evidence of recurrence after surgery. The patient was not started on pharmacological treatment as she was asymptomatic following surgery.

Appendiceal Crohn's disease is not infrequent. However, this case generated interesting learning points due to the unusual disease manifestation in our context. The atypical morphological appearance of excessively swollen oedematous appendix should raise suspicion of another aetiology.

## 4. Conclusion

We described a rare presentation of isolated appendiceal Crohn's disease as acute appendicitis in an older South Asian female. The atypical morphological appearance of the appendix should raise suspicion of Crohn's disease. This case highlights the importance of histopathological analysis of the specimen, especially in abnormal clinical findings. The prognosis of such patients seems to be good, and additional treatment is rarely needed.

## Figures and Tables

**Table 1:** Summary of case series and reports on appendiceal Crohn's disease diagnosed following appendectomy for acute appendicitis.

No.	Author	Year	Region	Sample	Further tests	Further treatment	Follow-up
1	Yang	1966-1977	Michigan, USA	14	Colonoscopy	1: resection of ascending colon, 1: total colectomy, 1: ileal and caecal resection	Other patients: asymptomatic on long-term follow-up
2	Ariel	1986	Israel	20	Colonoscopy	None	No recurrence
3	Prieto-Nieto	1975-1995	Madrid, Spain	10 (0.2%)	Colonoscopy	1: excision of enterocutaneous fistula	No recurrence
4	Akbulut	2006-2010	Diyarbakir, Turkey	18 of 5262	Not reported	Not reported	Not reported
5	Emre	2009-2012	Malatya, Turkey	6 of 1255 showed granulomatous inflammation	Negative for TB	Not reported	Not reported
6	Yokota	2010	Japan	1	Colonoscopy	None	No recurrence
7	Han	2007-2013	Seoul, South Korea	12 of 2179	Colonoscopy, AFB and Tb-PCR negative	None	No recurrence
8	El-Saady	2016	Egypt	1	Colonoscopy	None	No recurrence
